# Automated deep learning-based AMD detection and staging in real-world OCT datasets (PINNACLE study report 5)

**DOI:** 10.1038/s41598-023-46626-7

**Published:** 2023-11-09

**Authors:** Oliver Leingang, Sophie Riedl, Julia Mai, Gregor S. Reiter, Georg Faustmann, Philipp Fuchs, Hendrik P. N. Scholl, Sobha Sivaprasad, Daniel Rueckert, Andrew Lotery, Ursula Schmidt-Erfurth, Hrvoje Bogunović

**Affiliations:** 1 Department of Ophthalmology and Optometry, Medical University of Vienna, Vienna, Austria; 2 Christian Doppler Lab for Artificial Intelligence in Retina, Department of Ophthalmology and Optometry, Medical University of Vienna, Vienna, Austria; 3 Institute of Molecular and Clinical Ophthalmology Basel, Basel, Switzerland; 4 Department of Ophthalmology, University of Basel, Basel, Switzerland; 5 NIHR Moorfields Biomedical Research Centre, Moorfields Eye Hospital NHS Foundation Trust, London, UK; 6 BioMedIA, Imperial College London, London, UK; 7 Institute for AI and Informatics in Medicine, Klinikum rechts der Isar, Technical University Munich, Munich, Germany; 8 Clinical and Experimental Sciences, Faculty of Medicine, University of Southampton, Southampton, UK

**Keywords:** Macular degeneration, Retinal diseases, Computer science

## Abstract

Real-world retinal optical coherence tomography (OCT) scans are available in abundance in primary and secondary eye care centres. They contain a wealth of information to be analyzed in retrospective studies. The associated electronic health records alone are often not enough to generate a high-quality dataset for clinical, statistical, and machine learning analysis. We have developed a deep learning-based age-related macular degeneration (AMD) stage classifier, to efficiently identify the first onset of early/intermediate (iAMD), atrophic (GA), and neovascular (nAMD) stage of AMD in retrospective data. We trained a two-stage convolutional neural network to classify macula-centered 3D volumes from Topcon OCT images into 4 classes: Normal, iAMD, GA and nAMD. In the first stage, a 2D ResNet50 is trained to identify the disease categories on the individual OCT B-scans while in the second stage, four smaller models (ResNets) use the concatenated B-scan-wise output from the first stage to classify the entire OCT volume. Classification uncertainty estimates are generated with Monte-Carlo dropout at inference time. The model was trained on a real-world OCT dataset, 3765 scans of 1849 eyes, and extensively evaluated, where it reached an average ROC-AUC of 0.94 in a real-world test set.

## Introduction

Since the introduction of optical coherence tomography (OCT)^1^, and its commercialization in 1996^2^, this fast and non-invasive scanning technology has been in widespread use in clinical studies and routine clinical practice, especially in the field of ophthalmology. A variety of eye diseases can be diagnosed and monitored using the high-fidelity 3D volumes produced by OCT with its micrometer resolution. In tandem with this development, the standard clinical diagnosis for common macular diseases such as diabetic macular edema (DME) and age-related macular degeneration (AMD) has revolutionized and is now based primarily on OCT scans^3–6^.

For the management of AMD, the leading cause of blindness in elderly patients, the cross-sectional view of the macular region offered by 3D OCT, compared to 2D color fundus photography, provides important insight into the staging of AMD. Currently, AMD is classified into early/intermediate (iAMD), and the two late stages neovascular AMD (nAMD or wet AMD), and geographic atrophy (GA), based on the presence of drusen, exudation, and outer retinal atrophy on color fundus photographs, respectively^7^. Furthermore, OCT is invaluable for guiding the treatment of nAMD with anti-VEGF agents^4,8–11^. It is expected that OCT will bring a similar value in the future for monitoring of GA, now that the first complement inhibition agents have become available^12–15^. Thus, it is of paramount importance to accurately stage the disease in an AMD patient, and detect the conversion from iAMD to nAMD and/or GA immediately upon its onset to initiate timely treatment and maximize preservation of visual function in patients^11,16–19^.

Numerous OCT scans are acquired daily, as part of the management of patients with AMD in a busy clinical routine. This in parallel creates large-scale real-world imaging repositories within the picture archiving and communication systems (PACS) of hospitals. Such repositories, with their large amount of retrospectively and prospectively collected, yet unstructured data, are a treasure trove of information that, once curated, can be successfully mined for clinical insights about AMD progression and its pathomorphological risk factors. However, this defines a new challenge for the curation of such large unstructured unlabeled data repositories for statistical and machine learning analysis^20–23^.

Artificial intelligence (AI), with the advent of deep learning, has offered a path toward creating automated image recognition systems using convolutional neural networks (CNNs) that were shown capable of automated diagnosis at the level of retinal experts^24^. There has been an abundance of prior work on OCT classification^25–31^ and segmentation^24,32–36^. However, the majority of those works addressed only a 2D image classification task, primarily focusing on the classification of central OCT cross-sectional slice (B-scan). Therefore, only a minority of the works addressed the task of classification and staging of 3D OCT volumetric scans of patients with AMD^37–43^.

In this paper, we propose a deep learning method to determine if the current OCT scan shows biomarkers corresponding to the early/intermediate stage of AMD^7,44^ or if the eye has already converted to one of the two late stages of the disease, namely nAMD or GA^7,13,45^. To achieve the goal of automated AMD staging on datasets extracted from real-world clinical cohorts, we propose a two-stage deep learning approach to classify macula-centered 3D OCT volumes to detect the presence of four different biomarkers, i.e., macular neovascularization (MNV), macular atrophy (MA), drusen and normal retina as defined below. To the best of our knowledge, this work represents the first approach to classify and stage the entire 3D OCT volume of AMD patients with scans acquired in a real-world clinical setting.

Our main contributions can be summarized as follows:

- The development of a flexible and effective deep learning model to classify real-world OCT volumes.
- A proposed pipeline that exploits the key benefits of our approach, B-scan level classifications combined with uncertainty quantified volume predictions, to efficiently group, clean, grade, and curate large amount of retrospective OCT datasets.
- The extensive evaluation of the network on a real-world hold-out test set and the external evaluation on a large-scale real-world test set under image domain and population shift.

## Materials and methods

### Participants and imaging characteristics

A real-world dataset (denoted as PINN) consisting of volumetric OCT scans and the associated electronic health record (EHR) was collected from the retrospective branch of the PINNACLE consortium^46^. It was extracted from the University Hospital Southampton, and the Moorfields Eye Hospital, UK and contains data of patients over 50 years old and diagnosed with AMD in the period of (2007–2019). The PINN therefore represents twelve years of clinical routine at two large UK eye hospitals. It consists of a total of 156,872 OCT volumes of 6,616 patients acquired with Topcon scanners (Topcon Corporation, Tokyo, Japan): spectral domain (SD)-OCT T-1000 and T-2000, or the swept source (SS)-OCT Triton.

These scans were complemented with an in-house graded dataset (denoted as OPT) of Topcon scans (199 OCT-Volumes), available at the OPTIMA Lab at the Medical University of Vienna, Austria. This dataset is extracted from a repository of clinical study data and was included to represent prototype, clear-cut cases with only one stage-defining AMD biomarker present, in contrast to the mixture of biomarkers typically co-occurring in the real-world PINN scans.

The image resolution and size of the Topcon scans differed in all three image dimensions, and we restricted ourselves to the most common scan mode present in the datasets, i.e. 128 B-scans, each with 512 A-Scans, denoted as *y* and *x* dimension respectively, covering a $20^{\circ }\times 20^{\circ }$ field of view. The axial, *z* dimension, varied from $z=480$–650 for the T-1000, $z=885$ for the T-2000, and $z=992$ for the Triton Topcon device. The qualitative comparison of image quality between Topcon models and the distribution of scans with different *z* dimensions can be found in the Supplement (Table S1 and Fig. S1).

This study adhered to the tenets of the Declaration of Helsinki and the standards of Good Scientific Practice of the Medical University of Vienna. The retrospective data collection from the clinical sites in the UK has been approved and the need for informed consent was waived by the Wales Research Ethics Committee 4, UK (ref. 19/WA/0079). The presented study is part of the PINNACLE initiative and was approved by the Ethics Committee of the Medical University of Vienna, Vienna, Austria (ref. 1246/2016).

### Data curation and grading

To obtain the reference labels from PINN for training, validation and testing of our supervised deep learning approach, we split the grading into three steps. In brief: (1) a subset of scans was selected based on EHR, (2) the subset was manually cleaned to remove bad quality scans and scans not centered at the macula, and (3) the grading of the training set by an experienced non-retinal specialist, and the validation and test sets by a retinal specialist.

**Figure 1:**
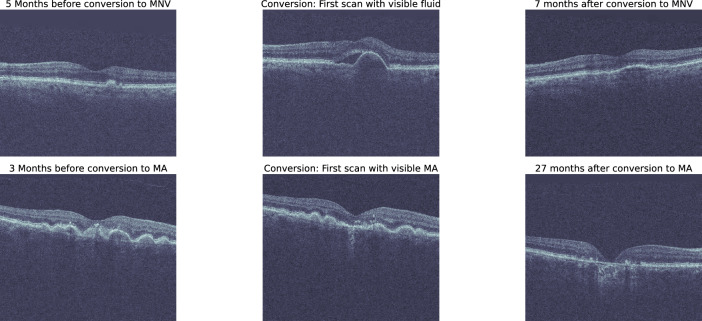
Example of two eyes in the PINN dataset that convert from early/intermediate to a late stage AMD. Top row: Eye with small drusen, which 5 months later shows a visible occurrence of retinal fluid. Bottom row: Eye with large drusen, which 3 months later shows a visible occurrence of atrophy.

Based on the EHR, we determined an approximate scan label for a randomly selected but disease stratified subset of the PINN dataset. We only included fellow eyes of those eyes that were undergoing treatment with anti-VEGF, with a few additional eyes representative of a healthy retina. This selection criteria increased the chance of encountering recently converted late-stage cases (Fig. 1). The resulting subset was manually cleaned, curated, and labeled by a computer scientist with experience in retinal image analysis (non-medical grader). Cleaning involved removing scans of non-AMD diseases, bad quality scans, and scans without biomarkers considered to be related to the AMD stages. The cleaning was a necessary step for an efficient grading process, as the EHR were not detailed enough to derive the biomarkers visible on a scan, and the long follow-up and daily clinical routine scans give rise to unavoidable scan quality issues. We relied on the presence of drusen, fluid, and atrophy as representative imaging biomarkers of iAMD, nAMD, and GA, respectively. In the following we will describe the presence and absence of these three biomarkers with the binary labels of DRUSEN, MNV (Macular Neovascularization) and MA (Macular Atrophy) and the absence of all three of them as NORMAL. B-scan examples of each biomarker are shown in Supplementary Fig. S1, while the corresponding clinical descriptions of these labels can be found in the grading protocol (Supplementary material).

Such a curated set, together with OPT, is denoted as the main dataset (MDS). It consists of 3995 OCT volumes from 2079 eyes of 1963 patients (Supplementary Fig. S3) containing scans with biomarkers of iAMD, nAMD and GA AMD at the B scan level, and hence also at the volume level, simultaneously.

### Deep learning-based OCT volume classification model

**Figure 2:**
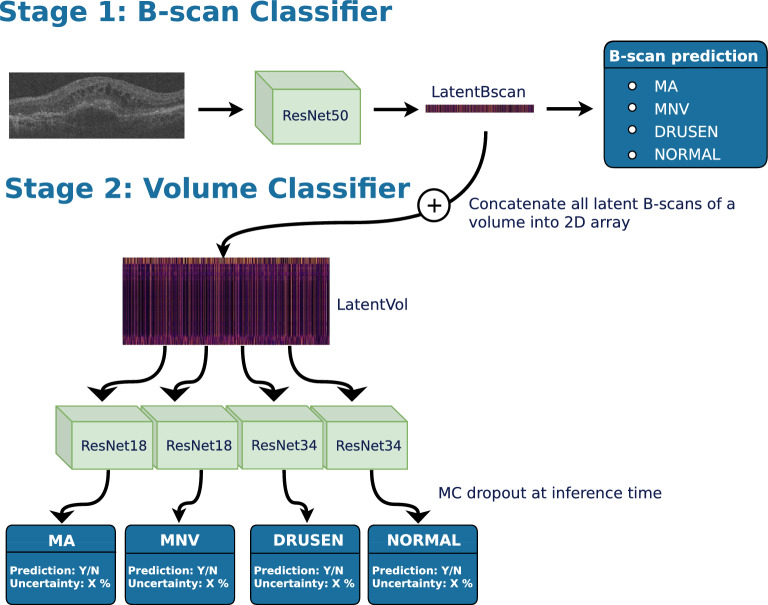
The proposed deep learning model consists of the following two CNNs: (i) B-scan classifier, which predicts the presence of biomarkers at each individual B-scan, and (ii) Volume classifier, which collates the features learned from the B-scan classifier to obtain a prediction at the OCT volume level for macular atrophy (MA), active nAMD (MNV), DRUSEN and NORMAL.

We implemented a two-stage deep learning model, composed of two 2D CNNs that are used to train a B-scan classifier, and an OCT volume classifier, respectively (Fig. 2). Such an approach allows us to learn from datasets with variable label granularity (B-scan-level vs. volume-level). In addition, it avoids using fully 3D CNNs that have much larger number of parameters and are more difficult to train with limited volume-level labels available.

**Figure 3:**
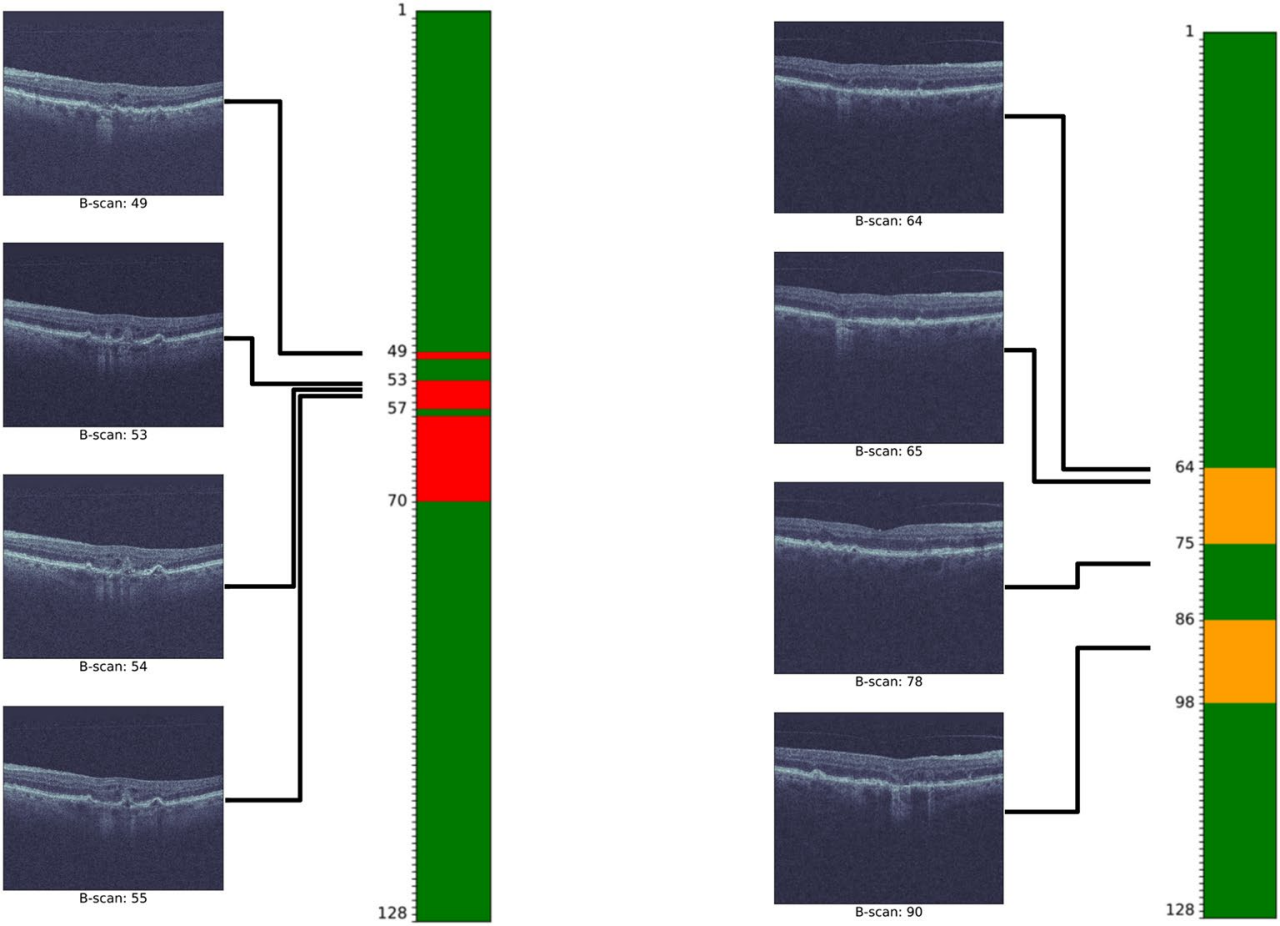
Example of B-scan localisation result on the test set. A volume predicted as MNV (first column) and a volume predicted as MA (second column). B-scan-level classification is displayed on top with MNV in red, MA in yellow, and green otherwise, with four illustrative B-scans, seven true positive examples, and one true negative example.

The first stage consists of an ImageNet-pretrained ResNet50 that is trained to identify disease categories on a B-scan. This stage serves two purposes. First, it provides a B-scan-wise encoding in the form of a 2048D vector (*LatentBscan*). Second, it enhances the interpretability of the model by offering a B-scan-level localisation of the predicted class within the volume (Fig. 3). By concatenating all the volume’s LatentBscan representations into a 2D tensor, we obtain a volume-level representation (*LatentVol*).

The second stage is trained independently of the first stage and takes the LatentVol as input with the size of $2048\times$#Bscans. It consists of four separate ResNets that are trained to perform a binary classification task at the level of an OCT volume, i.e., to predict if a particular disease-specific biomarker is present somewhere in the volume. ResNet18 is employed for predicting the presence of MA and MNV, and ResNet34 for predicting the presence of DRUSEN or if a volume is NORMAL.

#### Classification uncertainty estimation

The uncertainty estimates for each volume-level class are generated with Monte–Carlo dropout at inference time^47–50^ with ten evaluations per OCT scan. The uncertainty is quantified using the margin of confidence metric (MoC), based on the fact that we have several binary problems and the observations described in Milanés-Hermosilla et al.^51^. As stated above, we have four binary problems that reflect the presence and absence of the biomarkers in the OCT volume. Each run in Monte Carlo dropout inference equips us with a softmax output between 0 and 1 for the positive and negative class. The MoC estimates the uncertainty of a binary problem by subtracting the lower softmax value from the higher one, i.e. the more likely value, per inference run and averages them over the total amount of Monte-Carlo dropout runs. Further computational details can be found in Milanés-Hermosilla et al.^51^. All ResNets were derived from the standard PyTorch^52^ ResNet implementation by adding a dropout layer and a linear classification head.

#### Training details

Both stages of the model were trained with weight decay, dropout and label smoothing. The first stage B-scan model was trained with a SGD optimizer and a cyclic learning rate, while the second stage volume model was trained with the Adam optimizer^53^. For Stage 1, the pretrained ImageNet^54^ weights of the B-scan model were initialized for the Topcon model by further pretraining on the KERM dataset^26^ for 100 epochs. After that, we trained the Topcon B-scan model for 300 epochs on the MDS B-scan dataset (Supplementary materials). In both pre-trainings standard data augmentations (random rotations, cropping, erasing, and horizontal flipping) were applied and the B-scans were resized to $224 \times 224$ px , using the default PyTorch resize function with bilinear interpolation. For Stage 2, the LatentVol embedding was created once for the entire training, validation and test sets. Hence, the first stage B-scan-level CNN can be seen as frozen, while we trained and tuned in the second stage four different ResNets, for each binary biomarker, in the lower-dimensional embedding without additional data augmentation for 300 epochs. Different ResNets were trained with respect to batch-size, learning rate and depth (18, 32, 50). The final ResNet types for the four volume-level models were chosen based on the performance on the validation set, measured by the sum of their sensitivity and specificity.

## Evaluation methodology

### Internal dataset for OCT classification

In the following, we describe the OCT volume-level dataset that we used for training, validation, and testing of our model. The dataset used to train the B-scan-level classifier is described in the Supplementary material.

To serve as a training set, a total of 3765 scans from 1733 patients and 1849 eyes were randomly selected from MDS, and were graded using the grading rules (Supplementary material) by an experienced non-medical grader, and therefore are considered as silver-standard labels.

To serve as validation and hold-out test sets, a subset of MDS scans was chosen at random in a label-stratified manner by eye and disease according to the limited EHR available. The validation and test set, both consisting of distinct 115 OCT volumes from 115 eyes of 115 patients per set, were then graded by a retinal specialist for the presence of DRUSEN, MNV, MA or absence of these biomarkers (NORMAL). Out of these 230 scans, 44 had to be excluded during grading due to poor scan quality, disease not being AMD, non-active MNV, or scans centered at the optical nerve head instead of the macula. The final validation set consisted of 90 volumes, and the test set of 96 volumes that were patient independent between each other. In particular, the test set consists of 96 distinct eyes from 96 distinct patients. Table 1 shows the distribution of biomarkers in those three sets.

**Table 1:** Distribution of the classes (types of biomarkers present) of OCT volumes in the training, validation and test sets. Note that OCTs, patients and eyes may be counted more than once as several biomarkers can be present in the same OCT volume. In contrast, the last row, Total, contains the total amount of unique patients, eyes and OCT volumes used in the dataset splits.

Training and validation sets				Test set	
Biomarker present	Patients	Eyes	OCT-volumes	patients/eyes	OCT-volumes
MNV	1067	1123	1355	26	26
MA	341	344	1969	49	49
DRUSEN	658	688	735	85	85
NORMAL	113	117	132	6	6
Total	1733	1849	3765	96	96

### Internal evaluation and comparison to baseline methods

We report the volume-level performance of our model on the hold-out test set, where we predict the biomarker presence, for each of the four targets. The performance of the B-scan classifier is reported in the Supplementary materials.

We want to show that our approach reaches state-of-the-art performance while benefiting from the disease B-scan localization capability and simple to train setup. Therefore, we trained and evaluated two collections of ResNet3Ds, one ResNet3D trained for each biomarker with the standard PyTorch architectures ResNet3D34 and ResNet3D50, as baseline methods. The first network ResNet3D, was trained by tuning the learning rate, batch size, ResNet depth, i.e. 34 or 50, and also the size of the input (number of B-scans and slice resolution) via a hyperparameter search. The second network, denoted ResNet3DNaive, only uses 9 B-scans from each volume with a fixed B-scan size ($224\times 224$) and only the learning rate, model depth, and batch size were tuned. The B-scans were selected in both approaches by including the central B-scan $\pm X$ in each direction with a period of 2% of the B-scans available, e.g. with $X=4$ in the case of the second network we get the B-scan percentages: 41%, 43%, 45%, 48%, 50%, 52%, 55%, 57%, 59%. In both approaches, we trained one binary classifier per biomarker (One-vs-All) for 200 epochs. The operating points for both networks were determined by the maximum Youden-Index *J*^55^ on the PINN validation set.

To compare our model against the baseline approaches, we chose the following standard performance metrics: area under the ROC curve (AUC), balanced accuracy (BACC), accuracy (ACC), F1-Score, sensitivity and specificity. In addition, we report Matthews correlation coefficient (MCC), which has recently been shown to be a more truthful metric for binary classification problems with respect to the confusion matrix categories^56^, and is defined as

$$\begin{aligned} \text {MCC}=\frac{\text {TP} \cdot \text {TN} - \text {FP} \cdot \text {FN}}{\sqrt{(\text {TP}+\text {FP})\cdot (\text {TP} +\text {FN})\cdot (\text {TN}+\text {FP})\cdot (\text {TN}+\text {FN})}}, \end{aligned}$$

where TP, TN, FP and FN are the numbers of true positives, true negatives, false positives and false negatives, respectively.

### Evaluation of the classification uncertainty

In order to find a suitable threshold for distinguishing between certain and uncertain predictions, we optimized for the Matthews correlation coefficient (MCC). We compute the MCC per biomarker in the PINN validation set and use these derived MoC thresholds (i.e., MNV: 0.8, MA: 0.85, DRUSEN: 0.8, NORMAL: 0.5) to determine high-confidence predictions.

We verify the uncertainty estimates of our prediction by comparing the performance on three different subsets of the hold-out test set on the task to distinguish scans in the late stage (presence of MNV and/or MA) and non-late stage (no presence of MNV and/or MA). In particular, we will denote the “high confident” subset (HCTestSet) as the set of scans in the test set that have a high confidence prediction label (i.e. greater equal than the threshold defined above) simultaneously for MNV and MA. The “confident” subset (CTestSet) is defined similarly, but we only require that only one of these biomarkers is predicted with high confidence.

We also added three different naive B-scan approaches in this experiment, based on our first stage of the model only. The first model, GTCBScan, uses only the ground-truth label of the central B-scan to distinguish between late and non-late AMD. If the central B-scan was graded into MA or MNV, this approach “predicts” a late-stage scan. The two other models, denoted BScanModel, predict an OCT volume late if a single B-scan is predicted late, and the second one predicts late if at least $n\%$ of the total amount of B-scans are predicted as MNV or MA. We use $n=10$ as proposed in^29,57^, and $n=15$ for comparison. We report statistical significance between the groups using the non-parametric Mann-Whitney-Wilcoxon test.

### External validation on public datasets: Duke and UK Biobank

We also evaluated our model on an external public Duke dataset that used a different OCT scanner^58^. This dataset contains scans from 269 AMD (in early/intermediate and late stage according to our definition above) and 115 healthy eyes acquired with a Bioptigen OCT (Bioptigen, Inc. Research Triangle Park, NC). The Bioptigen scans were acquired with a field of view of ($6.7\,\text {mm} \times 6.7$ mm), size $(x, y, z) = (1000, 100, 512)$, and physical voxel size of 6.7 $\upmu$m $\times$ 67 $\upmu$m $\times$ 3.24 $\upmu$m, which was different than our internal development and evaluation sets from the Topcon-2000 scanner with a field of view of ($6\,\text {mm} \times 6$ mm), size (512, 128, 885), and a physical voxel size of $11.719 \,\upmu$m $\times$ $46.875\,\upmu$m $\times$ 2.6$\upmu$m. In order to report sensitivity, specificity and accuracy on this dataset, we randomly selected 10% of the total data and identified the operating point using the Youden-Index^55^ and applied it on the remaining 90% of the data. We report the same metrics as above for the task of distinguishing healthy vs AMD scans.

To further investigate the screening properties of our approach under population and scanner domain shift, we applied the network to the UK Biobank^59^ dataset (UKBB). The UKBB consists of 175,869 lower quality Topcon-1000 OCTs from 85,709 patients with a variety of diseases, where the majority is expected to be healthy, representative of population screening. We divide our analysis into two parts. First, we screened the total amount of OCT volumes for late-stage AMD, i.e., for volumes with dry and wet late-stage AMD. Second, we extracted the OCT volumes of all patients above 75 years of age, which resulted in 137 volumes of 74 patients, and screened them again for late-stage AMD.

The two cohorts were filtered to remove low quality scans by applying a quality index (QI) based thresholding per B-scan, proposed by Stein et al.^60^. After applying the filter, 121,608 OCT volumes from 71,763 patients remained from the first cohort and 63 scans from 45 patients from the second cohort. Then both subsets of UKBB were classified by our model and uncertain positive late-stage predictions, MoC smaller than 0.6, were ignored to adjust for the scanner model domain shift. Our classifier detected 621 scans with MA and/or MNV in the first cohort and 3 scans in the second cohort. To evaluate our classification in the first cohort, all positive predicted scans, i.e. with MNV or MA, were graded by a retinal specialist for the presence of MNV, MA secondary to AMD or other exudative retinal diseases such as diabetic macular edema (DME), retinal vein occlusion (RVO), central serous chorioretinopathy (CSS) or atrophy secondary to other degenerative retinal diseases. Therefore, we report only the positive predictive value (PPV). The second cohort, of patients above 75, was graded in the same manner but in its entirety, to be able to report sensitivity and specificity, in addition to PPV.

## Results

### Internal evaluation and comparison to baseline methods

**Figure 4:**
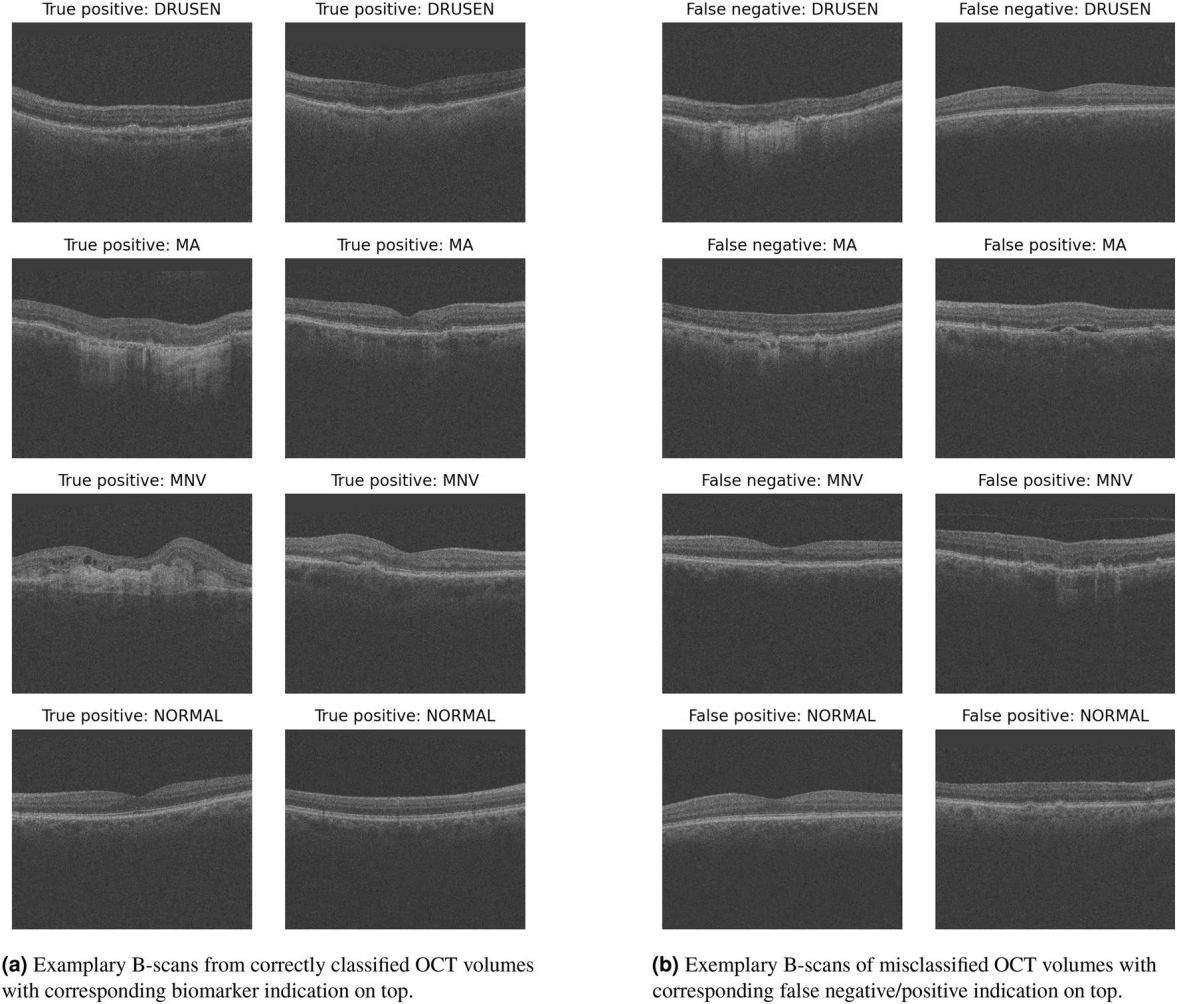
Examples of correct and misclassified OCT volumes from the internal PINN test set with relevant B-scan displayed.

**Figure 5:**
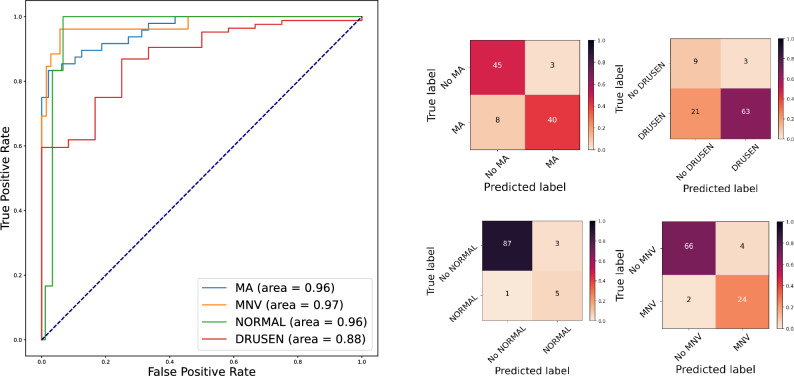
ROC-curves with ROC-AUC (area) and confusion matrices for detecting each biomarker.

Qualitative examples of correct and misclassified OCT volumes are shown in Fig. 4. ROC-curves and confusion matrices of our model per biomarker are shown in Fig. 5. Our model reached very high ROC-AUCs on the test set across all biomarkers (0.96–0.97), with the exception of DRUSEN (0.88). On the one hand, the performance for DRUSEN corresponds to the fact that our test set also contains early AMD cases that show only minimal drusenoid elevation of the Retinal Pigment Epithelial (RPE) on one single B-scan. This makes it particularly hard to distinguish between normal curved retinas, noise, and real DRUSEN and leads to false negative cases. The false positive cases on the other hand are detected in scans with rather small examples of Pigment Epithelial Detachment (PED), partly vanished RPE because of atrophy, or in scans with large amounts of retinal fluid that obscure the shape of the retinal layers and their structures. As discussed above, our test set contains scans with several biomarkers, hence one biomarker can obscure the presence of the other biomarkers. Late stage AMD scans can show large retinal deformation by fluid, SHRM, or RPE debris. These conditions can increase the difficulty of detecting small drusen or atrophic areas with small diameter.

Comparison to baseline approaches averaged across biomarkers is given in Table 2. The metrics per individual biomarker are in the Supplementary Table S4. Although our model outperformed both baselines for the biomarkers MNV and NORMAL, the model detected slightly fewer MA cases than the fully tuned ResNet3D approach. The performance for the DRUSEN biomarker is mixed for all three approaches, indicating a challenging test set. Overall, our approach showed comparable or better performance compared to baselines with an additional benefit of B-scan-level disease localization. While slightly behind in BACC, our approach achieves a higher MCC, indicating a better correlation between the true label and the prediction.

**Table 2:** Comparison of our two-stage approach with two baseline ResNet3D classification models. Columns: ROC-Area under the curve (ROC-AUC), balanced accuracy (BACC), accuracy (ACC), Matthews correlation coefficient (MCC), F1 Score, sensitivity and specificity per model. Bold marks the highest value.

Model	ROC-AUC	BACC	ACC	MCC	F1 score	Sensitivity	Specificity
ResNet3D	**0**.**95**	**0**.**885**	0.839	0.628	0.752	0.842	**0**.**928**
ResNet3DNaive	0.914	0.84	**0**.**891**	0.619	0.8	**0**.**86**	0.82
Ours	0.944	0.867	0.883	**0**.**67**	**0**.**831**	0.835	0.899

### Evaluation of the classification uncertainty

The strategy to categorize the test set volumes, as described above in the evaluation methodology section, into certain and uncertain cases by thresholding the MoC value leads to 28 and 68 out of 96, respectively, for high confident and confident prediction labels by excluding uncertain cases based on two criteria as described above. In particular, we measure the metrics below on these two subsets of scans and compare them to the metrics computed on the whole test set. The approach based on the ground-truth central B-scan labels performs poorly, as expected in this task. The sole B-scan model approach with 1 B-scan and 10% thresholds of late predicted scans perform better in terms of sensitivity with a trade-off of higher false positive rates. As these metrics are highly volatile with respect to the percentage thresholds, we plot the MCC for different thresholds (Supplementary Fig. S4). All metrics increase to nearly perfect score for the high confident labeled subset (only 1 false negative) and increase for the confident subset substantially (only 3 false negatives) compared to the whole dataset. The detailed metrics are in Table 3.

**Figure 6:**
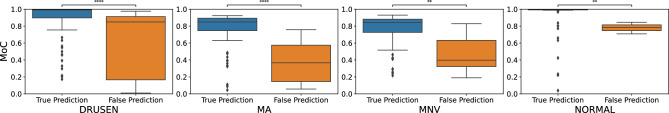
Margin of confidence (MoC) of true and false predictions for the four biomarkers. Statistical significance: **denotes $p<0.01$, and ****denotes $p<0.0001$.

The uncertainty MoC values and their corresponding prediction in the test set are shown in Fig. 6. MoC values were significantly different between correct and false prediction for each biomarker. This observation points to the direction that the MoC can be used to identify reliably classified samples.

**Table 3:** Performance of models on the binary classification task: late AMD vs non-late AMD on the volume level. The GTCBScan model uses only the central B-scan grading of the retinal expert. The BScanModel uses only the first stage of our network with different thresholds. The thresholds define how many B-scans have to be classified as late AMD in a volume so that the volume itself is classified as late. “Ours” corresponds to our full model on different subsets of the test set of PINN. CTestSet corresponds to the subset of scans from TestSet with certain prediction, and HCTestSet to the subset of scans with high certainty.

Model	Subset	Volumes	Threshold	BACC	ACC	MCC	F1 score	Sensitivity	Specificity
GTCBScan	TestSet	96	1	0.757	0.76	0.52	0.759	0.76	0.694
BScanModel	TestSet	96	1	0.859	0.896	0.762	0.925	**0**.**969**	0.75
BScanModel	TestSet	96	10%	0.906	0.906	0.796	0.928	0.906	0.906
Ours	TestSet	96	N/A	0.914	0.896	0.792	0.917	0.859	0.969
Ours	CTestSet	68	N/A	0.966	0.956	0.91	0.965	0.932	**1**.**0**
Ours	HCTestSet	28	NA	**0**.**975**	**0**.**964**	**0**.**919**	**0**.**974**	0.95	**1**.**0**

### External validation on public datasets: duke and UK Biobank

On the external Duke dataset, we reached an almost perfect ROC-AUC of 0.994 in the task of distinguishing AMD vs. healthy eyes, compared to the 0.992 reported in the original work^58^ and 0.97 to 0.999 in more recent works^40,43,61^. This result shows that our model has an ability to generalize to other OCT devices. Furthermore, the high performance on this external test set (BACC 0.98) compared to the performance on our internal PINN test set (BACC 0.9) shows the level of difficulty of our real-world datasets compared to clinical study datasets.

On the external UKBB, the classifier achieved a low PPV for the presence of active MNV (0.14) and a high PPV for the presence of MA (0.88) on the first cohort of all OCT volumes. Reformulating the classification target to the presence of retinal fluid (nAMD, RVO, CSS, DME,...) the model achieved a PPV of 0.89 for retinal fluid, and 0.91 for fluid-and/or-atrophy vs. no-fluid-and-no-atrophy on this cohort. In addition, if we ask to detect scans without mixing of the two late stage biomarkers, i.e., with only retinal fluid and no atrophy, and scans with atrophy and no fluid, we then see a rise in the performance to a PPV of 0.94 for the detection of atrophy, and a PPV of 0.99 for retinal fluid. In the second cohort of patients above 75, we detected 1 out of 1 atrophy and 1 out of 2 cases of retinal fluid, which leads to a sensitivity/specificity of 1.0/1.0 for atrophy and 0.5/1.0 for retinal fluid. These results suggest, although trained solely on an AMD dataset with the vast majority of scans from different OCT devices, that our model can detect general retinal fluid and atrophy, and hence has learned the biomarker itself and not AMD specific features.

## Discussion

Numerous OCT datasets are collected in eyecare centers around the world, with OCT being the gold standard imaging modality for managing AMD patients. Although many studies developed and applied AI to retrospectively investigate OCT study imaging data, only a limited amount of these examine and exploit real-world OCT data from the clinical routine. This is partly due to EHR being unstructured and not offering the diagnostic granularity that can be used to extract the patient subcohort of interest. Therefore, we proposed a deep learning classification pipeline that is able to detect the current disease stage on a given OCT scan of an AMD patient from a real-world longitudinal dataset. The availability of the AMD stage in the datasets can then be used to perform downstream clinical and statistical analysis with a larger number of images, boosting the value of retrospective studies of OCT scans.

We presented a robust and flexible approach to classify retrospective AMD datasets into the three main stages of the disease. We achieved a good performance with ROC-AUC of 0.944, averaged over the biomarkers, in a randomly selected and retinal expert-graded independent test set. In addition, this work was trained and tested nearly entirely on real-world data, and we showed that our uncertainty estimates can subgroup our test set and reflect the quality of the prediction by Monte Carlo dropout at inference time. Although many 2D classification and segmentation approaches were proposed to stage AMD on the B-scan level, the more practical and more relevant classification of the whole volume in real-world data has received less prior attention. Furthermore, many of the proposed deep learning methods^37–42,62^ and the classical machine learning methods^63,64^ for 3D classifications were trained and evaluated solely on clinical study data. Unfortunately, the well-specified patient inclusion criteria for such studies do not reflect the challenges of a real-world clinical dataset, where several AMD stage-associated biomarkers can be present in the same OCT volume and sometimes even on the same B-scan. Additionally, quality issues of OCTs taken in the daily clinical routine are not manifested in most clinical study datasets. In particular, we used a limited amount of training labels to create a robust classifier directly from retrospective data extracted from two different hospitals.

Our proposed method for analyzing volumetric OCT data is general and modular, where the underlying CNNs can be interchanged and retrained separately. The two-stage classification approach has two main advantages over existing 3D classification approaches: First, we can use different datasets to train the B-scan and volume-level classifier. Since the grading on a B-scan level is much less involved than on volume-level, new scans can be labeled rather fast and with already existing 2D labeling strategies. This allows us to adapt to before unseen scan noise patterns from different OCT scanner types rather easily by leveraging transfer learning and a smaller amount of newly graded B-scans. In addition, most progress in image classification is focused on 2D images, which are easier to train and can be implemented with less computational costs. This also means that we can easily interchange DL architectures and pretrained models depending on their availability and progress in the field of 2D classification. And second, simultaneously, we receive a B-scan-level explainability of the volume classification as it is based on the B-scan classification that indicates the B-scans from where the disease label was likely identified. This can be used, in combination with the uncertainty estimate, to decide and guide clinicians to review only specific regions of the OCT volume that are deemed especially relevant for volume-level staging.

We chose to focus our classification on the most representative biomarkers that correspond to the main stages of AMD. In particular, we consider scans with some form of drusen (see definition below), in the absence of late-stage biomarkers, to be in the early/intermediate stage. Scans with visible intraretinal cystoid fluid or subretinal fluid were considered to be in the active nAMD (wet AMD), i.e. MNV, and we identify the onset of the GA stage of AMD, i.e. MA, with a visible smaller atrophic lesion, i.e. complete RPE and Outer Retinal Atrophy (cRORA) with at least 250 $\mu$m (micrometer) diameter. Although the presence of a biomarker on one B-scan could imply a volume-wide label, this approach is error-prone in real-world data. In particular, as the biomarkers can be sparsely spread out over the volume and when only a few B-scans are considered, they can be missed. This can be observed in the Supplementary Fig. S4, where different number of B-scan predictions of the 3D volume were used to predict the stage of the whole volume. Even in our cleaned test set, with no bad quality scans, this approach shows a highly volatile performance with respect to the number of B-scans chosen.

Our study has several limitations. The used datasets were all acquired with some variant of Topcon OCT scanner, and hence the trained models may not generalize to other popular devices, e.g. Cirrus and Spectralis. Nevertheless, we did show good generalization to the Bioptigen device, so it is expected to perform similarly on scans with such signal-to-noise ratio. Although the Bioptigen device differs quite substantially in resolution, see the validation section for more details, we point out that the overall appearance of the B-scan slides is more similar to Topcon compared to e.g. a Heidelberg Spectralis device. This is partly due to their comparable light source characteristics with half-bandwidth (53.7 nm vs. 50 nm) and wavelength (860 nm vs. 840 nm) and the lack of B-scan averaging. In general, to ensure a wider generalizability, domain shift adaptation approaches^65–67^ should be pursued. Another limitation is that we restricted our focus to datasets of AMD patients that have been determined as such from the EHR. This lowers the probability for the presence of non-AMD biomarkers. Although not trained to detect other disease biomarkers, a negative NORMAL classification label, in combination with the absence of positive MA, MNV and DRUSEN predictions, should also indicate the presence of diseased OCT scans in a general population cohort beyond AMD. The approach by Araújo et al.^68^ to out-of-distribution (OOD) detection could be a possible mitigation for this aspect of disease screening in a general population. This is not a strong limitation for applications, as the grouping of patients at the level of AMD and non-AMD through EHRs is quite effective even with unstructured health records. However, our results on the UKBB reflect the strengths and caveats of population-biased training of machine learning models. Although trained to detect MNV and MA secondary to AMD, the classifier was able to detect fluid and atrophy when exposed to a screening population beyond the eyes with AMD.

A promising application of our work is for the retrospective detection of the onset of the stage itself. We can use the acquired labels at the volume level to automatically label the longitudinal OCT datasets into early/intermediate and late stage of the disease. Observing the appearance of the biomarkers over time allows us to use the onset of drusen to characterize the early/intermediate stage until a late stage is reached by either development of fluid or atrophy. A patient typically will convert to a late stage with either fluid first or atrophy first visible on an OCT. Hence, a patient who converts to the late stage via fluid presence can start to be considered as a nAMD (wet AMD) case, even if there is no fluid present later on due to treatment with anti-VEGF injections. In contrast, a patient that develops atrophy after the early/intermediate stage can be considered to be in a GA stage until the possible onset of fluid. Thus, we can make use of the volume label to provide the expert graders with the timelines (Supplementary Fig. S5), which indicate the most likely conversion point, from normal to early/intermediate, and to late, leading to efficient grading and detection of the onset of the two main late AMD stage, in longitudinal OCT data. The MoC uncertainty of the prediction can be used in this setting to extract especially challenging and interesting cases. Although this usage of the uncertainty falls more in the domain of the data engineering part of gradings, it can be seen as a first step to test the computed MoC and evaluate further its application in a real-world screening setting that can assist retinal specialists and clinicians in their daily routine to judge the quality of the results presented by a potential general screening algorithm. Thus, be seen as a blueprint for a general cleaning and grading pipeline for real-world data analysis of medical image data and can help to leverage the unused potential of large retrospective datasets.

### Supplementary Information

Supplementary Information.

## Data Availability

The datasets generated during and/or analysed during the current study are not publicly available due to privacy constraints. However, the data may be available from the Medical University of Vienna subject to local and national ethical approvals. Please contact hrvoje.bogunovic@meduniwien.ac.at for any requests.

## Code availability

The machine learning model components described and applied in this work were available as part of the PyTorch library.
