# Supplementary material for: Automated deep learning-based AMD detection and staging in real-world OCT datasets (PINNACLE study report 5)
Source: Sci Rep. 2023 Nov 9;13:19545. doi: 10.1038/s41598-023-46626-7 (PMC10636170; doi:10.1038/s41598-023-46626-7)
Supplement: Supplementary file 1 — Supplementary Information. [file 41598_2023_46626_MOESM1_ESM.pdf]

Automated deep learning-based AMD detection and staging in real-world OCT datasets (PINNACLE study report 5)

Oliver Leingang, Sophie Riedl, Julia Mai, Gregor S. Reiter, Georg Faustmann, Philipp Fuchs, Hendrik P. N. Scholl, Sobha Sivaprasad, Daniel Rueckert, Andrew Lotery, Ursula Schmidt-Erfurth, and Hrvoje Bogunović

Supplementary material

Imaging characteristics

The number and the characteristics of the OCT volumes in the PINN dataset are provided in Table S1 with a qualitative comparison of a representative sample shown in Figure S1.

Scanner Type	z-Dim	Number of Volumes
T-2000	885	132259
T-1000	480	17841
Triton	992	5494
T-1000	650	1278

Table S1. Number of OCT volumes with the axial (z-Dim) dimensions and scanner type in the PINN dataset.

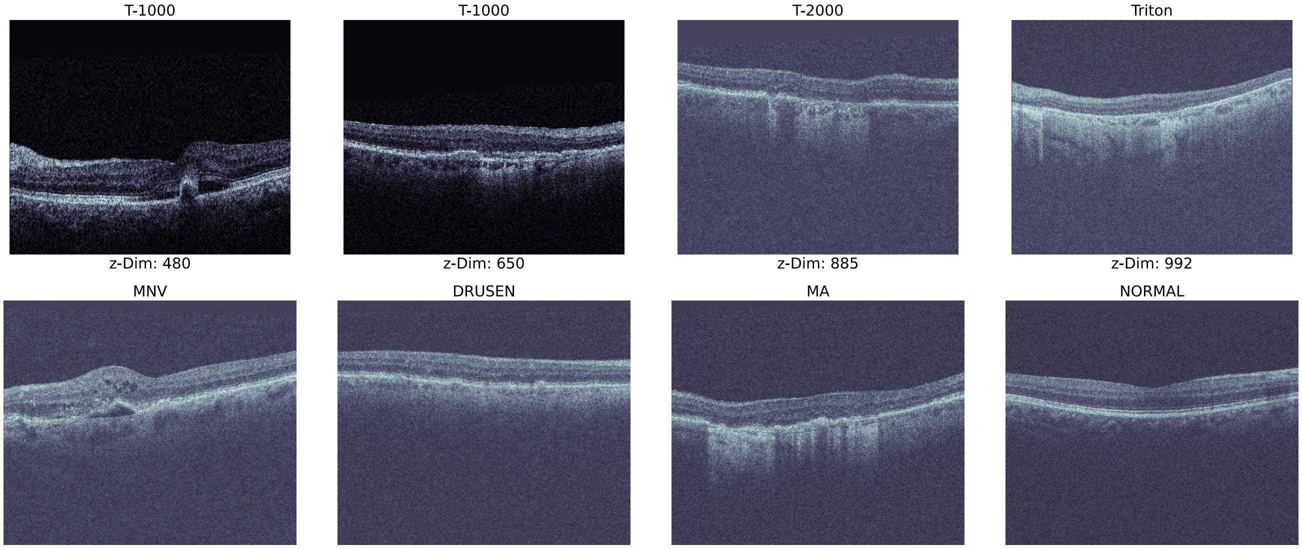

Figure S1. Top row: Qualitative comparison of image quality and resolution in our datasets consisting of Topcon T-1000, T-2000, and Triton, with their axial (z-Dim) dimension below and scanner type on top. Bottom row: Examples of B-scans with the corresponding biomarker label on top.

Grading protocol

The grading on OCT volume level was performed using an in-house developed web application designed to grade OCT volumes in an efficient manner with the help of hotkeys and digital caliper capabilities.

Gold-standard labels:

The validation and test data were graded by a retinal expert using the following criteria to label a B-scan:

- MNV: presence of either PED or SHRM together with either IRF or SRF, based on Metrangolo et al.[45].
- DRUSEN: presence of at least one drusenoid elevation of the RPE.
- MA: cRORA as defined by presence of all the following three features in an axially overlapping manner over an extent of more than 250µm: choroidal hypertransmission, RPE attenuation or loss, evidence of overlying photoreceptor degeneration, based on Sadda et al.[13].
- NORMAL: healthy appearing retina, presence of an epiretinal membrane (ERM) was tolerated.

Silver-standard labels:

The training data was graded by an experienced non-medical grader with relaxed rules as follows:

- MNV: a clear presence of either IRF or SRF.
- DRUSEN: a clear presence of at least one drusenoid elevation of the RPE.
- MA: a clear presence of atrophy as defined by presence of all of the following three features in an axially overlapping manner over an extent of more than 150µm: choroidal hypertransmission, RPE attenuation or loss, evidence of overlying photoreceptor degeneration.
- NORMAL: healthy appearing retina without visible distortions and deformations of the retinal layers.

As soon as one B-scan of a volume was graded into one of the biomarkers MNV, DRUSEN, and MA the whole volume was assigned this label. The grading was independent for biomarker, i.e. a volume can simultaneously have the labels MNV and DRUSEN. NORMAL label was determined by the absence of these three disease-related biomarkers.

B-scan-level classification

Training set A total of 106,892 2D B-scans acquired with Spectralis OCT (Heidelberg Engineering, Heidelberg, Germany) from a publicly available dataset[26], supplemented with 7,829 B-scans showing atrophy from an internal dataset, were used to pre-train the B-scan model. This dataset is denoted as KERM. The Topcon training set for the B-scan classifier itself, consisted out of 2,967 B-scan slices from 1,059 OCT volumes from 358 patients and 372 eyes extracted from the volume level training set of MDS described above. The B-scans were graded by an experienced non-medical grader into MNV, DRUSEN, MA and NORMAL and can therefore be considered silver standard labeled. Please refer to Table S2 for more details.

Validation and Test set The B-scans for the validation and test set were extracted in a deterministic way (5 B-scans per volume on percentage y-positions 30%, 46%, 50%, 54% and 70%) from the respective volume validation and test set of MDS and graded by a retinal expert for the presence/absence of the four biomarkers (MNV, MA, DRUSEN and NORMAL). Please see Table S2 for the distribution of biomarkers on these B-scans.

Training and validation					Test		
Biomarker present	Patients	Eyes	OCT-Volumes	B-Scans	Patients/Eyes	OCT-Volumes	B-scans
MNV	233	237	325	765	22	22	65
MA	170	172	557	1169	50	50	158
DRUSEN	191	194	352	909	71	71	188
NORMAL	47	47	47	643	24	24	69

Table S2. B-scan characteristics in the development set (training and validation sets) (left) and test set (right).

Results The classification performance at the B-scan level is reported in Table S3. Examples of B-scans from PINN test set and their classification is shown in Figure S2.

Biomarker	ROC-AUC	BACC	ACC	MCC	F1 Score	Sensitivity	Specificity
NORMAL	0.935	0.898	0.895	0.702	0.902	0.903	0.894
DRUSEN	0.841	0.823	0.845	0.671	0.842	0.724	0.923
MA	0.960	0.937	0.942	0.863	0.942	0.924	0.950
MNV	0.979	0.955	0.961	0.850	0.962	0.946	0.963

Table S3. ResNet50 classification performance on the B-scan level test set.

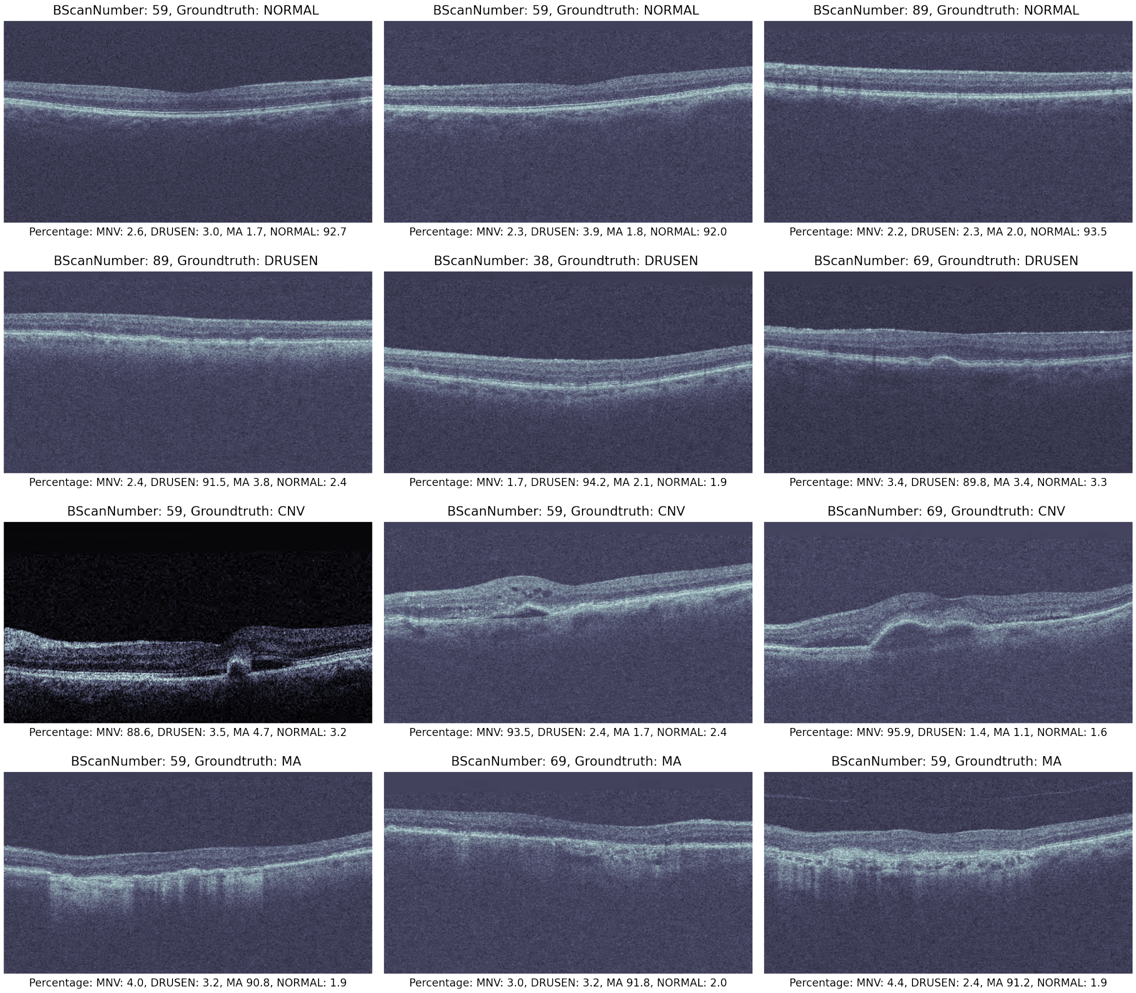

Figure S2. Example B-scan classification on the test set. B-scan number and the ground-truth label are displayed on top, while the softmax output (%) for each class is displayed below. Each row corresponds to a different ground-truth biomarker presence.

Supplementary Tables and Figures

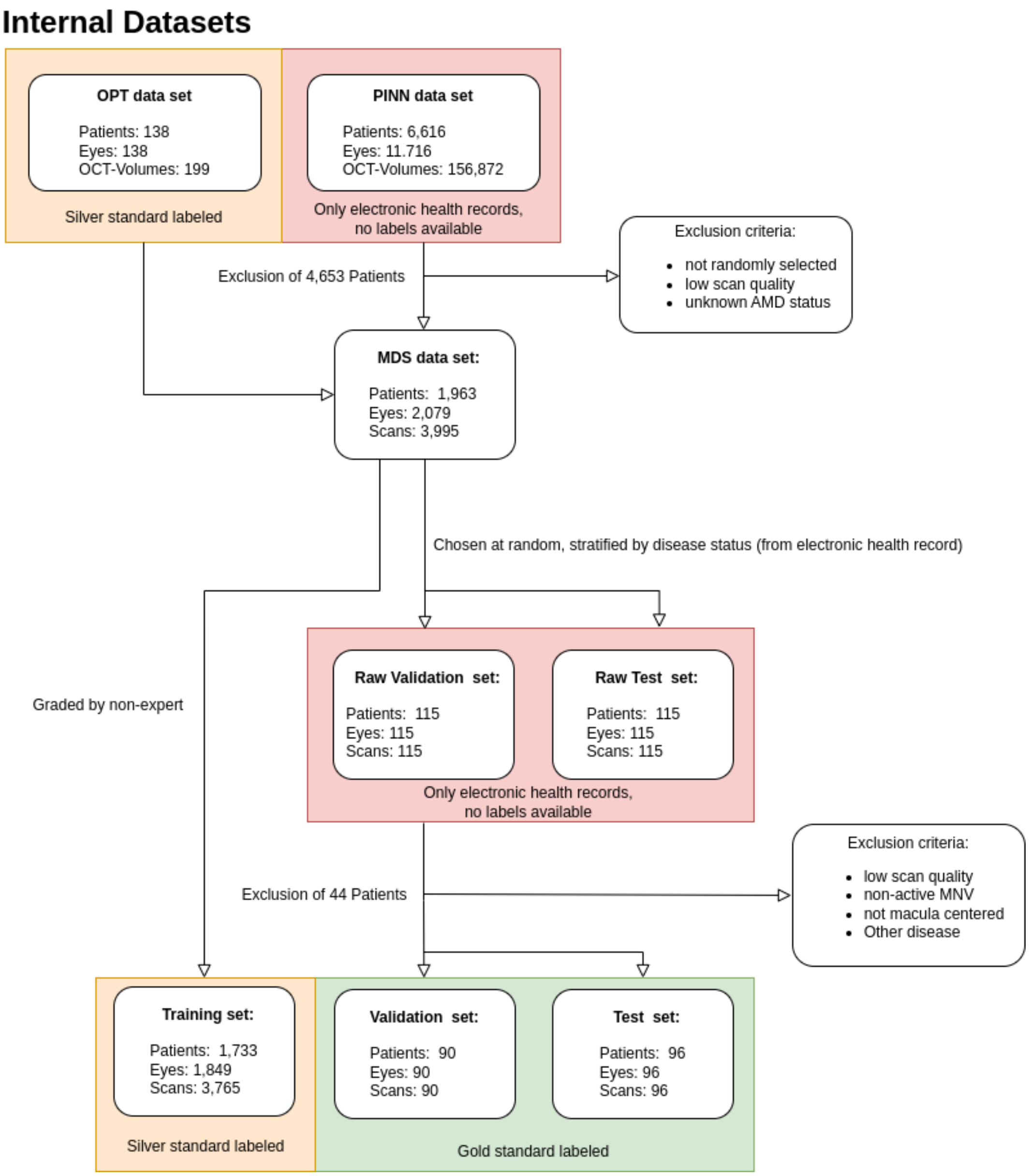

Figure S3. Data diagram: Shows the combination of datasets and the selection used for training, validation and testing of the network. Orange is used as color-code for labels graded by an non-retinal expert, red for only electronic health record labels and green for retinal expert graded labels.

Model	ROC-AUC	BACC	ACC	MCC	F1 Score	Sensitivity	Specificity
**MNV**							
3D	0.956	0.873	0.885	0.722	0.8	0.846	0.9
3DNaiveBSL	0.903	0.849	0.885	0.707	0.784	0.769	0.929
Ours	**0.975**	**0.933**	**0.938**	**0.847**	**0.889**	**0.923**	**0.943**
**NORMAL**							
3D	0.95	**0.939**	0.885	0.557	0.522	**1.0**	0.878
3DNaiveBSL	**0.972**	0.883	0.927	0.583	0.588	0.833	0.933
Ours	0.965	0.9	**0.958**	**0.701**	**0.714**	0.833	**0.967**
**DRUSEN**							
3D	**0.933**	**0.812**	0.667	**0.399**	0.768	0.624	**1.0**
3DNaiveBSL	0.809	0.702	**0.823**	0.33	**0.896**	**0.859**	0.545
Ours	0.875	0.75	0.75	0.357	0.84	0.75	0.75
**MA**							
3D	0.963	0.917	0.917	0.834	0.917	0.898	0.936
3DNaiveBSL	**0.973**	**0.926**	**0.927**	**0.858**	**0.932**	**0.98**	0.872
Ours	0.961	0.885	0.885	0.775	0.879	0.833	**0.938**

Table S4. Comparison of our approach with two different 3D models in the task to detect the presence or absence of the four biomarkers MNV, NORMAL, DRUSEN and MA at a volume-level in PINN dataset. Columns: ROC-Area under the curve (ROC-AUC), balanced accuracy (BACC), accuracy (ACC), Matthews correlation coefficient (MCC), F1 Score, sensitivity and specificity per model. Bold marks the highest value.

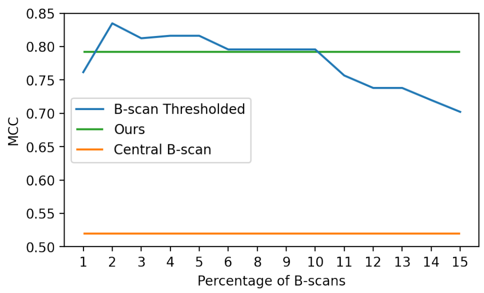

Figure S4. Comparison between B-scan predictions only for different %-thresholds of late stage predicted B-scans, expert graded central B-scan only prediction and our 2-stage approach on the whole test set. MCC on the y-axis and different %-thresholds on the x-axis.

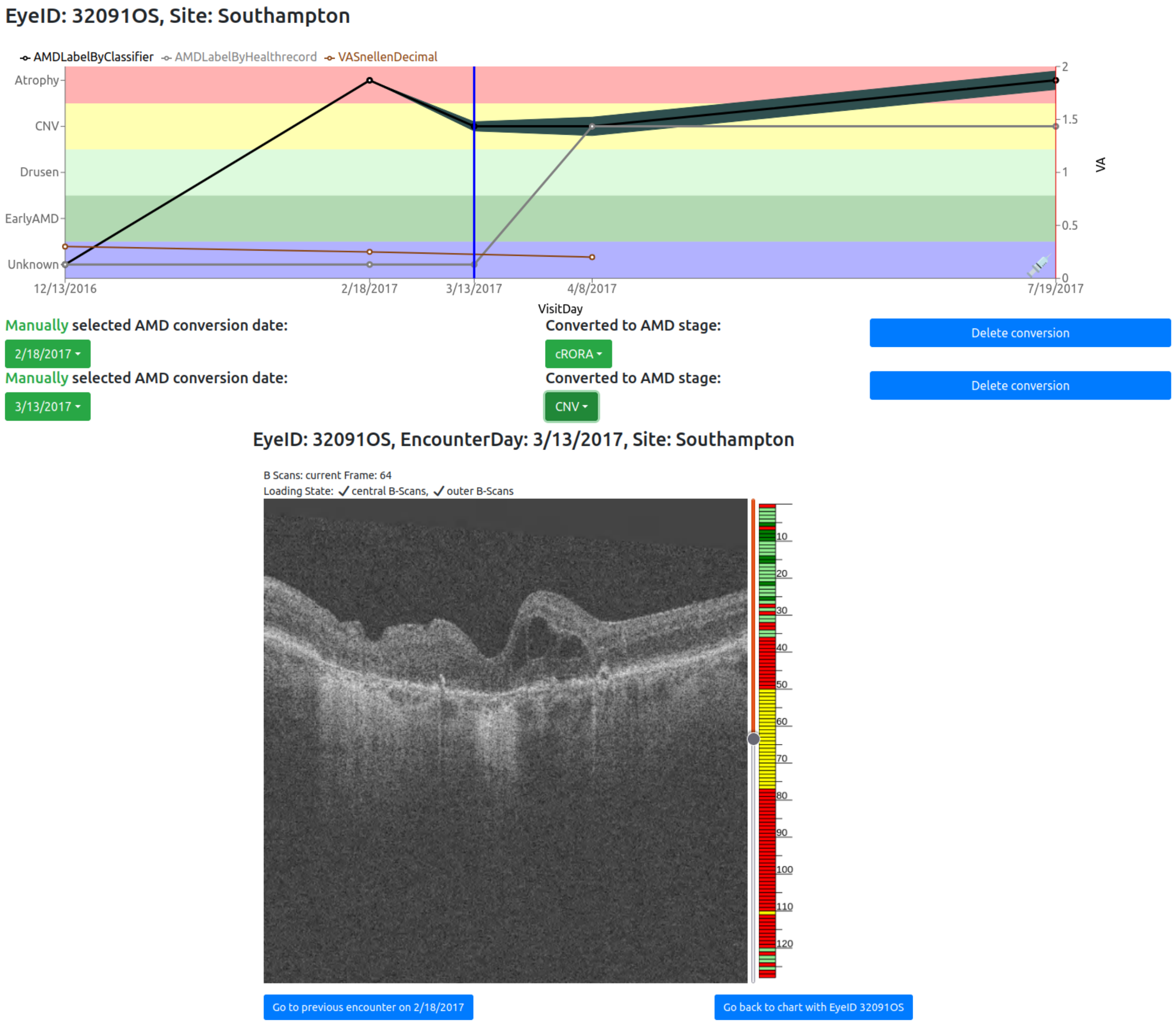

Figure S5. Example of conversion grading timeline (top) and OCT volume viewer with biomarker B-scan labels (bottom). Legend for the B-scan labels: Yellow: MNV, red: MA, green: NORMAL, light green: DRUSEN.
